# Association Between Anatomic and Clinical Indicators of Injury Severity After Moderate-Severe Traumatic Brain Injury: A Pilot Study Using Multiparametric Magnetic Resonance Imaging

**DOI:** 10.1089/neur.2023.0122

**Published:** 2024-03-13

**Authors:** Dmitry Esterov, Ziying Yin, Trevor Persaud, Xiang Shan, Mathew C. Murphy, Richard L. Ehman, John Huston, Allen W. Brown

**Affiliations:** ^1^ Department of Physical Medicine and Rehabilitation, Mayo Clinic College of Medicine, Rochester, Minnesota, USA.; ^2^ Department of Radiology, Mayo Clinic College of Medicine, Rochester, Minnesota, USA.; ^3^ Department of Mayo Clinic School of Graduate Medical Education, Mayo Clinic College of Medicine, Rochester, Minnesota, USA.

**Keywords:** biomarker, magnetic resonance elastography, magnetic resonance imaging, outcome, post-traumatic amnesia, traumatic brain injury

## Abstract

This study sought to identify whether an anatomical indicator of injury severity as measured by multiparametric magnetic resonance imaging (MRI) including magnetic resonance elastography (MRE), is predictive of a clinical measure of injury severity after moderate-severe traumatic brain injury (TBI). Nine individuals who were admitted to acute inpatient rehabilitation after moderate-to-severe TBI completed a comprehensive MRI protocol prior to discharge from rehabilitation, which included conventional MRI with diffusion tensor imaging (DTI). Of those, five of nine also underwent brain MRE to measure the brain parenchyma stiffness. Clinical severity of injury was measured by the length of post-traumatic amnesia (PTA). MRI-assessed non-hemorrhage contusion score and hemorrhage score, DTI-measured white matter fractional anisotropy, and MRE-measured lesion stiffness were all assessed. A higher hemorrhagic score was significantly associated with a longer length of PTA (*p* = 0.026). Participants with a longer PTA tended to have a higher non-hemorrhage contusion score and softer contusion lesions than the contralateral control side, although the small sample size did not allow for assessment of a significant association. To our knowledge, this is the first report applying MRI/MRE imaging protocol to quantitate altered brain anatomy after moderate-severe TBI and its association with PTA, a known clinical predictor of post-acute outcome. Future larger studies could lead to the development of prediction models that integrate clinical data with anatomical (MRI), structural (DTI), and mechanical (MRE) changes caused by TBI, to inform prognosis and care planning.

## Introduction

Moderate-severe traumatic brain injury (TBI) is a complex condition with a broad spectrum of injury severity, in which functional outcomes for survivors can range from total dependence to full recovery.^1^ Accurately predicting outcome can help determine prognosis and guide rehabilitation efforts. Clinical measures of injury severity, such as the duration of post-traumatic amnesia (PTA), have been found to predict outcome after moderate-severe TBI.^2-4^ However, the duration of PTA can range from minutes to several months,^5^ and by the time that accurate prognostication is feasible in some instances, patients may no longer be in the care of specialists who can discuss prognosis and planning for rehabilitation.^6^ Therefore, an objective indicator of injury severity that can be obtained in the hospitalization phase after moderate-severe TBI that provides clinically relevant, long term prognostic information would be useful to patients, their caregivers, and clinicians in discussions regarding recovery, outcome trajectories, and rehabilitative needs.^6^

Although its use in the acute phase after TBI may be increasing, the prognostic utility of brain MRI remains uncertain.^7^ Conventional MRI (relative to computed tomography scanning) allows for better detection of intraparenchymal lesions and brainstem injury, as well as shear injury representing diffuse axonal injury.^8^ In particular, brainstem lesions on MRI have been associated with poor global outcome 1 year after moderate-severe TBI.^7^ Advanced MRI techniques such as diffusion tensor imaging (DTI) can better understand the disruptions to the structural integrity of white matter (WM) fibers after moderate-severe TBI^9^ and have been found to correlate with functional outcomes.^10^ Yet despite advances in neuroimaging techniques such as DTI in assessing the structural changes in the brain, many aspects of TBI may remain undetected, such as changes in brain tissue biomechanics directly affected by an external mechanical force.

Magnetic resonance elastography (MRE) may serve as an invaluable complement to conventional MRI in the context of TBI, providing non-invasive, quantitative insights into the mechanical behavior of the brain and its mechanical environment.^11,12^ Brain MRE is unique as it allows for analyzing the induced brain motion in response to intrinsic or extrinsic excitation and by doing so, can assess quantitative measures of the mechanical properties of the brain non-invasively, which is not possible with other imaging modalities.^13^ By integrating MRE with conventional MRI with DTI, it may be possible to assess the anatomical, structural, and mechanical changes associated with TBI in a more comprehensive manner, to determine with greater depth the unique variance in injury characteristics after moderate-severe TBI. This could then be correlated with clinical outcomes more accurately than is currently possible by standard image classification methods. Brain stiffness has been reliably measured by MRE in healthy individuals,^14^ and has been studied in individuals with dementia as well as in those with brain tumors.^15–17^ However, there have been no prior studies assessing brain MRE in individuals with moderate-severe TBI.

This pilot clinical study sought to assess the feasibility of obtaining an objective indicator of injury severity using a multiparametric brain MRI – a combination of both conventional MRI with DTI and MRE – in individuals participating in acute inpatient rehabilitation after moderate-severe TBI, and analyze its relationship to the duration of PTA, which is a clinical measure of injury severity and a known predictor of functional outcome. In addition, we sought to obtain preliminary data on whether multiparametric brain MRI would correlate with several clinical outcomes 1 year after injury.

## Methods

### Participants

This feasibility study was approved by the Mayo Clinic institutional review board. Participants in this study were individuals with moderate-severe TBI who were enrolled into Mayo Clinic's TBI Model System Center (TBIMSC) National Database (NDB). Inclusion criteria for the TBIMS NDB include the following: (1) hospitalization with a moderate-to-severe TBI (i.e., PTA > 24 h, trauma-related intracranial neuroimaging abnormalities, loss of consciousness >30 min, Glasgow Coma Scale (GCS) in the emergency department of <13), (2) being at least 16 years old at time of injury, and (3) presenting to a TBIMSC-affiliated acute care hospital within 72 h after injury. In addition to eligibility for enrollment in the TBIMS NDB, exclusion criteria for this study included those with contraindications to MRI, and individuals with Ranchos Los Amigos IV (those confused and agitated), who were unable to safely be transported to an MRI scanner. Individuals who had an intracranial procedure including a craniotomy or craniectomy were excluded from the MRE portion of the imaging protocol. Although previous reports have found that the vibrational amplitudes used in MRE are below the European Union whole-body vibration limit and European Union Guidelines,^18^ this cautious approach was undertaken to preclude any unanticipated interaction between the distinctive vibrational characteristics of MRE and the structural modifications of the cranium after the surgical interventions.

### Clinical variables and outcomes

Demographic characteristics were obtained on all individuals at time of informed consent. Our primary outcome was the duration of PTA, which was calculated as the number of days between the TBI and the first of two occasions within a 72-h period in which the participant was fully oriented (> 76 on the Galveston Orientation and Amnesia Test,^19^ > 25 on the Orientation Log,^20^ or documentation of 2 days with consistent orientation within a 3-day period in the acute medical record via the GCS with no intervening days at less than full orientation).

Outcomes at one year were assessed and included the Functional Independence Measure (FIM), Cognitive and Motor subscales and the Disability Rating Scale (DRS), as well as the Glasgow Outcome Scale Extended (GOSE), all commonly used outcome measures in studies of individuals with moderate-severe TBI, with adequate validity and reliability.^21–23^

### MRI/MRE imaging

**FIG. 1.**
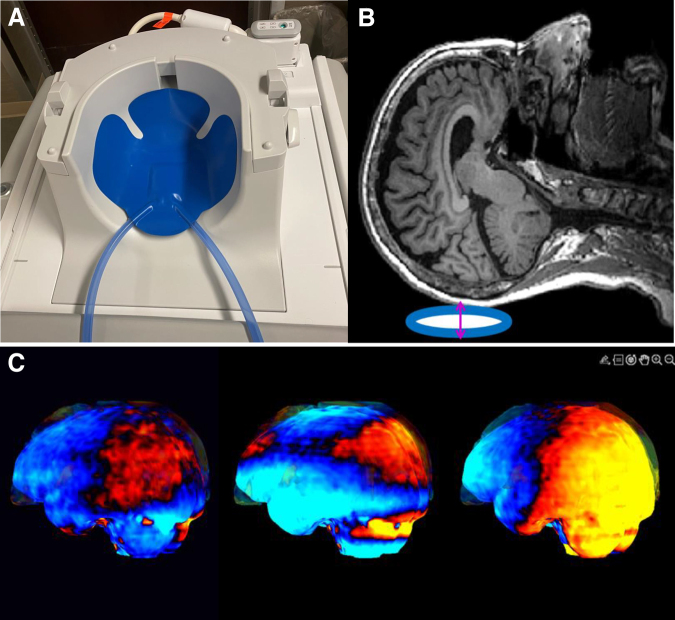
Overview of Brain magnetic resonance elastography (MRE) process: **(A)** Passive brain magnetic resonance elastography (MRE) driver positioned in magnetic resonance imaging (MRI) head coil. **(B)** Schematic representation of the soft pillow driver (shown in blue oval) positioned beneath the head, which generates vibrations in the anterior-posterior direction (illustrated by the pink arrow). **(C)** The resulting three-dimensional wave images, encoded across the x-, y-, and z-directions.

MRI/MRE were obtained while individuals were participating in rehabilitation on the acute rehabilitation unit, and performed when the medical care team determined that the participants could safely and comfortably undergo a 30-min scanning protocol. MRI/MRE were obtained using 3.0 T GE MRI scanners, and included a T1-weighted image, an axial T2-weighted image, an axial T2 fluid attenuated inversion recovery (FLAIR) image, an axial susceptibility-weighted angiography (SWAN) image, and a DTI scan. MRE scans involved the application of 60 Hz micrometer-level vibrations, generated by a commercially available pneumatic active driver (Resoundant Inc., Minnesota, USA). These vibrations were transmitted through a custom-made, soft, pillow-like head passive driver positioned beneath each participant's head to induce motion within the intracranial brain tissue (Fig. 1). Further details regarding imaging characteristics for this study can be found in Supplementary Data.

### Qualitative evaluation of MRI/MRE

Four MRI/MRE imaging-based predictors in this study were utilized to examine their correlation with clinical outcomes: MRI-assessed non-hemorrhage contusion score, MRI-assessed hemorrhage score, DTI-measured WM fractional anisotropy (FA), and MRE-measured lesion stiffness.

Assessment of pathology by conventional MRI was based on the methods of Avesta and coworkers.^24^ One board-certified neuroradiologist (J.H., with 34 years of experience) reviewed T1, T2, T2 FLAIR, and SWAN images of the enrolled patients, which were blinded for clinical information. The non-hemorrhage contusion score was determined by evaluating lesion sizes observed on FLAIR images, and it was categorized into three levels: (1) normal, (2) isolated lesion measuring <25% of the size of the corresponding structure, and (3) extensive lesion >25% of the size of the structure.^24^ The hemorrhage score was calculated by quantifying the number of lesions detected on SWAN images, and it encompassed five different scales based on the following criteria: (1) normal, 2) < 5 hemorrhagic foci, 3) between 5 and 50 hemorrhagic foci, and 4) > 50 hemorrhagic foci or hematoma.

The pre-processing of the diffusion data for DTI scanning involved employing a previously developed pipeline,^25^ encompassing procedures such as skull stripping, denoising, correction for head motion, eddy current distortions, Gibbs ringing, and Rician noise bias. Subsequently, the FA map was derived from the diffusion tensors by applying a non-linear least square fitting algorithm.^26^

**FIG. 2.**
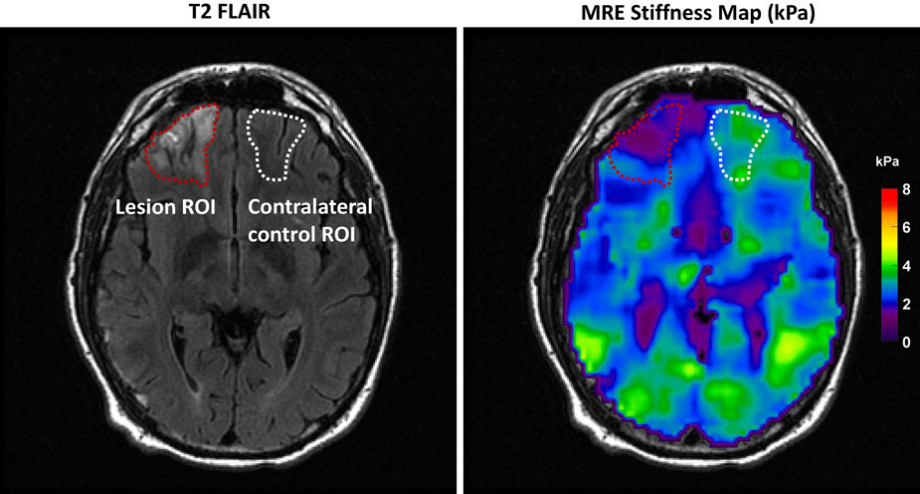
Examples of lesion region of interest and its contralateral control region of interest, delineated on the T2 fluid attenuated inversion recovery (FLAIR) image, and then registered and resampled to the magnetic resonance elastography (MRE) space.

For MRE analysis, the maps of brain stiffness were computed using a previously published neural network inversion algorithm.^27^ The neural network is trained using simulated inhomogeneous wave patches generated through a finite difference model. The FLAIR images were used to generate two regions of interest: lesion and contralateral normal brain. (Fig. 2). To account for any mechanical behavior not specifically caused by brain trauma, we calculated the ratio of lesion stiffness to the contralateral normal brain tissue stiffness. Further details relating to the quantitative evaluation of MRI and MRE can be found in the Supplementary Data.

### Statistical analysis

Continuous data were summarized as means and standard deviations (SD). Categorical data were summarized as counts and percentages. Spearman's correlation was utilized to examine the associations between between MRI/MRE indicators (non-hemorrhage contusion score, hemorrhage score, WM global FA, and the ratio of lesion to normal stiffness) and PTA. Multivariate analyses were foregone because of limited sample sizes. Although the PTA duration (in days) is inherently continuous, it was also categorized as an ordinal value, and dichotomized at 7 days, as PTA duration <7 days has been found to be reliable in predicting outcome.^3,5^ To compare MRI/MRE variables with PTA severity, non-parametric tests such as the Mann–Whitney *U* test or χ^2^ test were applied. However, no group comparison statistical analysis was conducted for MRE, given that only one patient with PTA >7 days had MRE scans. Statistical significance was defined as *p* < 0.05.

## Results

Nine patients were included in this study for MRI scans. Of these, three patients were excluded from the MRE portion of the protocol because of an intracranial procedure. Table 1 summarizes the demographic and clinical characteristics of the nine participants in this study. The mean age of the patient cohort at injury was 48.7 years (SD: 14.4, range: 34–74). Participants in the study were predominantly male (66.6%, 6 out of 9), and all of the participants were white. Participants had a mean length of stay in the acute hospital of 15 days (SD: 8.31, range: 6–30), and a mean length of stay of 12.3 days (SD: 6.89, range: 7–27) on the rehabilitation unit. Mean GCS on hospital admission was 9.89 (SD 5.51, range 3–15), mean days of PTA in the sample was 7.9 days (SD: 8.64, range: 1–21), and mean time to follow commands (TFC) was 1.78 days (SD: 2.05, Range: 0–7). MRI/MRE were obtained a mean of 24.89 days after injury (SD: 12.84, range: 12–50 days). All nine participants were evaluated for non-hemorrhage contusion score, hemorrhage score, and WM FA through MRI, whereas MRE scans were conducted for only six out of the nine participants, as the remainder had an intracranial procedure acutely after injury. The mean interval from injury to MRI was 24.9 days (SD: 13.6, range: 12–50).

**Table 1.** Demographic and Clinical Characteristics of Participants

***Participants***	***Sex***	***Race***	***Marital status***	***Education (years)***	***Age at injury (years)***	***GCS (days)***	***Penetrating TBI***	***Intracranial procedure***	***Acute hospital length of stay (days)***	***Acute rehabilitation length of stay (days)***	***PTA (days)***	***TFC (days)***	***Injury-to-MRI interval (days)***	***MRE***
1	Male	White	Married	12	39	14	No	No	19	9	8	1	26	Yes
2	Male	White	Married	12	45	15	No	No	8	9	1	1	12	Yes
3	Female	White	Married	16	53	9	No	No	12	7	3	2	17	Yes
4	Male	White	Married	16	74	15	No	Middle meningeal artery embolization	10	7	1	1	16	Yes
5	Female	White	Divorced	12	52	15	No	No	10	10	0	0	19	Yes
6	Male	White	Married	13	57	3	No	Craniotomy	26	27	21	2	44	No
7	Female	White	Single (never married)	16	26	3	No	No	6	8	2	1	13	Yes
8	Male	White	Single (never married)	12	34	3	Yes	Anterior skull base CSF leak repair with sphenoidotomy	30	20	21	1	50	No
9	Male	White	Married	12	58	12	No	Craniectomy	14	14	14	7	27	No

GCS, Glasgow Coma Scale; MRI, magnetic resonance imaging; MRE, Magnetic Resonance Elastography; PTA, post-traumatic amnesia; TFC, time to follow commands; TBI, traumatic brain injury; CSF, cerebrospinal fluid.

**FIG. 3.**
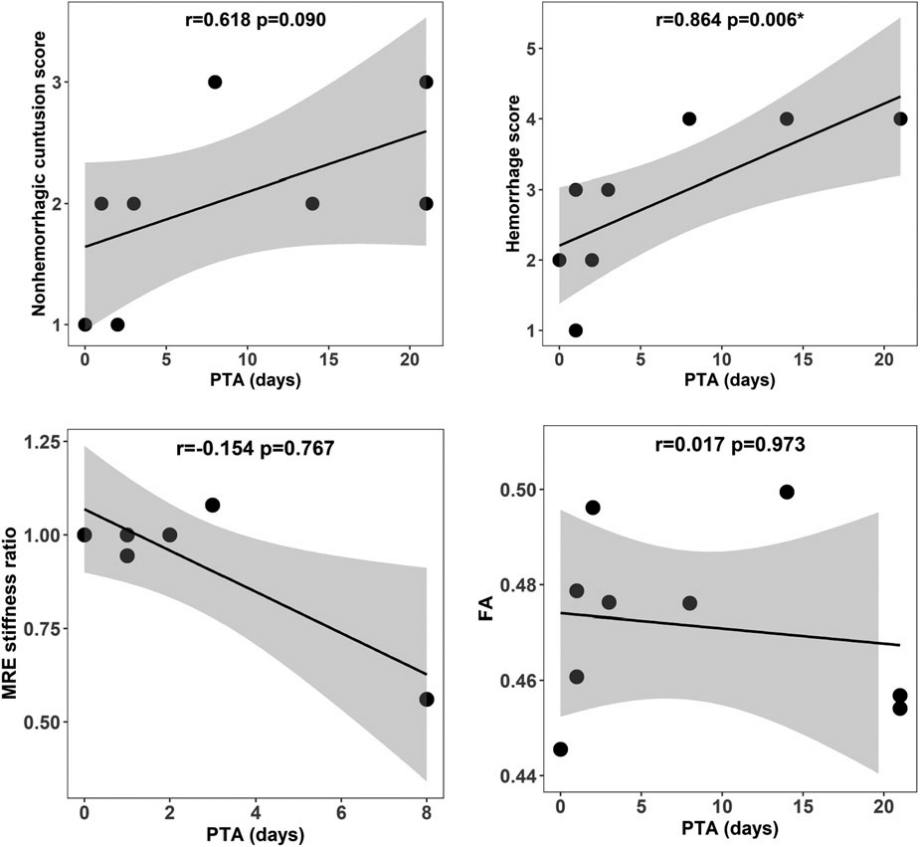
Spearman correlations between post-traumatic amnesia (PTA) days and magnetic resonance imaging (MRI)/magnetic resonance elastography (MRE) findings including hemorrhagic score, non-hemorrhagic contusion score, fractional anisotropy (FA), and MRE stiffness ratio.

**FIG. 4.**
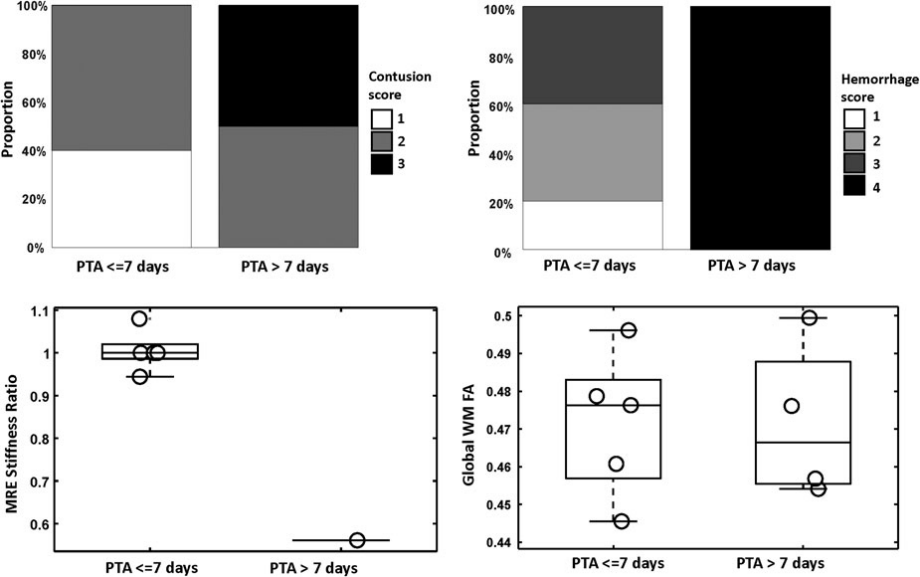
Correlation of magnetic resonance imaging (MRI)/magnetic resonance elastography (MRE) results with post-traumatic amnesia (PTA) >7 days. FA, fractional anisotropy.

Table 2 shows the neuroradiologist's evaluations of non-hemorrhagic contusion scores (as detected on FLAIR images) and hemorrhage scores (as detected on SWAN images), age-corrected global WM FA values, and the ratios of lesion-to-normal stiffness, all categorized by PTA severity. The correlations between the MRI/MRE findings with both PTA days and PTA severity are illustrated in Figures 3 and 4, respectively. Within the group of patients exhibiting PTA days ≤7 days, three out of five showed lower hemorrhage scores (with grades varying from 1 to 2), whereas every patient with PTA >7 days demonstrated the highest hemorrhage scores (grade 4). A significant correlation was observed between hemorrhage scores and PTA days (*p* = 0.006). The analysis also revealed a trend suggesting that patients with higher non-hemorrhage contusion scores (indicative of larger lesion sizes) are prone to experience prolonged PTA. However, this observation did not reach statistical significance. Individuals with PTA >7 days had a stastically significant difference in the number of days from injury to obtaining MRI (*p* = 0.016), as noted in Table 2, reflecting a greater time that it was determined participants with increased injury severity were medically stable to undergo the MRI/MRE scan.

**Table 2.** MRI / MRE Findings by Severity of PTA

	***PTA 0–7 days***	***PTA >7 days***	*p* ***value***
No. of participants with MRI	5	4	-
No. of patients with MRE	5	1	-
Injury-to-MRI interval (days) mean (SD)	15.4 (2.9)	38.8 (12.1)	0.016^*^
Contusion scores mean (SD)	1.60 (0.55)	2.50 (0.58)	0.15
Hemorrhagic score mean (SD)	2.20 (0.84)	4.0 (0)	0.016^*^
MRE lesion stiffness to contralateral side mean (SD)	1.0 (0.05)	0.56	-
Global WM FA mean (SD)	0.47 (0.02)	0.47 (0.02)	0.90

^*^ Statistical significance was defined as *p* < 0.05.

MRE, magnetic resonance elastography; MRI, magnetic resonance imaging; PTA, post-traumatic amnesia; SD, standard deviation; WM FA, white matter fractional anisotropy.

**FIG. 5.**
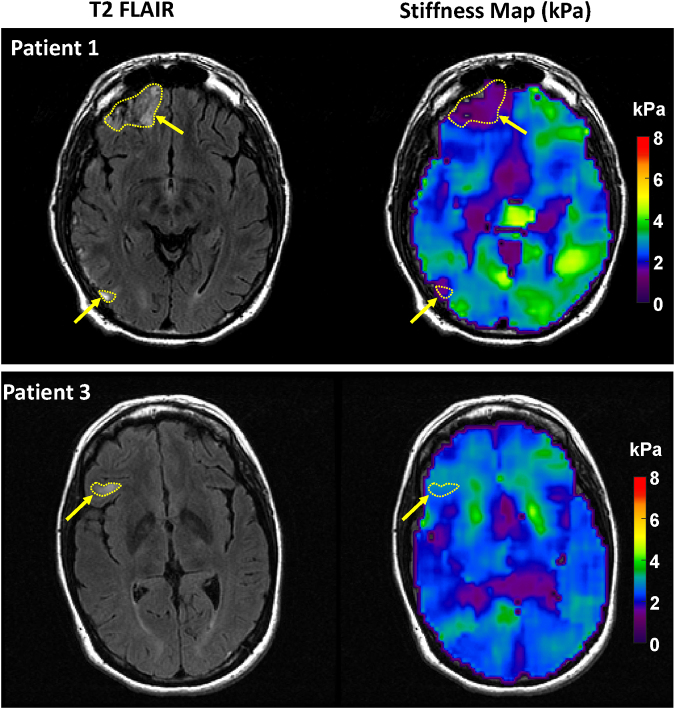
Magnetic resonance elastography (MRE) Examples. (Top) A 39-year-old male patient with post-traumatic amnesia (PTA) of 8 days. This patient exhibited multiple contusion lesions, characterized by significantly less stiffness when compared with the contralateral normal brain tissue. (Bottom) A 53-year-old female patient with PTA of 3 days. This patient also had multiple contusion lesions (with the largest one shown here via dotted line and arrow). Intriguingly, the lesion does not demonstrate significantly less stiffness than the corresponding contralateral region.

We identified a lower ratio of lesion-to-control tissue stiffness in patients with prolonged PTA. This indicates that within a contusion lesion, a reduction in stiffness (i.e., softer parenchyma than in the contralateral control region) could indicate more extensive and widespread brain tissue damage and influence subsequent recovery. Regrettably, the limited sample size within each group precluded the possibility of conducting a statistical analysis. Figure 5 displays examples from MRE, including representative patients with PTA durations of both less than or equal to 7 days and greater than 7 days. Participants with contusion lesions typically exhibit decreased stiffness relative to the contralateral control, a phenomenon that is particularly pronounced with the prolongation of PTA days. In this limited sample, no significant correlations were detected between WM FA values and PTA durations.

Out of nine participants, we obtained outcome data at 1 year for eight participants. Of those, the mean motor FIM score was 89 and the mean FIM cognitive score was 34.1. The maximum FIM motor score is 91, and the maximum FIM cognitive score is 35,^22^ indicating near full physical and cognitive independence in our participants. Two individuals scored 1 on the DRS, indicating mild disability, and the other participants scored 0, indicating no disability. Only one individual scored a 4 on the GOSE, and all other participants scored ≥6, indicating minimal disability. Prior studies have dichotomized GOSE based on either favorable outcomes of GOSE scores 4–8 or unfavorable outcomes of GOSE scores 1–3.^28^ Given overall favorable functional outcomes, further statistical analysis on 1 year outcomes was not performed.

## Discussion

We report a feasibility study of a multiparametric brain MRI in individuals participating in acute inpatient rehabilitation after moderate-severe TBI, and the first study to include MRE in relation to outcome after moderate-severe TBI. Given the known predictive utility of PTA as a clinical outcome measure, we analyzed the relationship between MRI-MRE and the duration of PTA. We found that in conventional MRI, there was a significant correlation between higher hemorrhagic scores and a longer duration of PTA. In addition, MRE findings showed a lower ratio of lesion stiffness in those with longer duration of PTA, indicating that decreased stiffness of brain parenchyma could be associated with clinical outcomes, although the small sample size did not allow for assessment of a significant association.

There have been several MRI studies that have correlated neuroimaging findings to PTA. In a retrospective study of 65 individuals including 45 with severe TBI enrolled in the TBIMS study and MRI performed for clinical purposes, traumatic microbleeds associated with traumatic axonal injury in the hippocampus and corpus callosum correlated with the duration of PTA.^29^ In another comprehensive, prospective MRI investigation of 490 patients with mild-to-severe TBI, findings indicated that both the location and the total quantity of traumatic axonal injury lesions, delineated by the number and volume of lesions on FLAIR and the number of lesions on T2-weighted gradient echo or susceptibility-weighted imaging, were associated with an extended duration of PTA.^30^ These findings are in alignment with our observations, demonstrating that the hemorrhage score (defined by the number of lesions in SWAN) is significantly correlated with the duration of PTA. It is noteworthy, however, that the injury location was not factored into our analysis, given the small sample size in our study. Additional details regarding the injury location for each patient are provided in Supplementary Tables S1 and S2.

This was the first clinical study of MRE in individuals with moderate-severe TBI. Of the five patients who completed MRE, a lower stiffness ratio (softer brain parenchyma) was present and associated with a prolonged length of PTA. This observation is novel, without available comparison data in humans. MRE has been studied in the past with healthy volunteers, and was found to demonstrate high test-retest reliability when measuring global brain stiffness as well as in subcorital gray matter.^14,31^ MRE has also been studied both as a biomarker for dementia,^15^ as well as for pre-operative assessment in those with meningiomas.^16^ In regards to TBI, MRE has been studied in animal models. Our findings are consistent with prior studies assessing TBI and MRE in animal models, including findings of decreased lesion stiffness ratio after mild TBI in rodents, as measured by ultrasonic shear wave elastography.^32^ Similarly, a significant decrease in the stiffness in the region of the injury was present in mice receiving a controlled cortical impact with both sham and naïve mice as controls.^33^ Potential causes for the reduction in stiffness was thought be caused by a reduction of cerebral blood flow, presence of edema, or hemorrhage. Further research is needed to determine the role that specific pathologies play in the reduction of tissue stiffness.

Notably, patients with longer PTA durations indicative of more severe injury experienced a significantly longer injury-to-scan interval than those with shorter PTA. This difference reflected a longer natural course of recovery for those with more severe injuries (reflected by PTA) and hence the need for more time to reach a stable condition suitable for MRI/MRE scanning. This time was reflected in both a longer acute hospital length of stay for these individuals, as well as in increased precautions in the acute rehabilitation setting to ensure safety (e.g., individuals still in PTA requiring additional support relating to acute medical rehabilitation needs as well as post-traumatic agitation). Despite the extended recovery periods, our findings show that these patients with longer PTA still exhibited higher hemorrhage and contusion scores, as well as more significant mechanical abnormalities, than those with shorter recovery times. This supports preliminary findings and is a signal of a potential significance of the proposed imaging biomarkers in evaluating brain injury severity.

Several studies have used DTI to better understand PTA after moderate-severe TBI. In one prospective study using DTI, the length of PTA was found to be associated with eight white matter neural structures related to cognitive functioning.^4^ We did not discern a significant correlation between global WM FA and extended PTA. This lack of correlation might be partially attributed to the limited sample size and restricted range of PTA, hindering the detection of such an effect, as well as potential bias from the FA estimation. This bias occurred during the process of age correction for FA, in reliance on previously published data, because of an absence of control data. Moreover, considering the substantial variability in the mechanism of injury and patient characteristics, coupled with the heterogeneity of the intersubject variations in pathology locations, future investigations should prioritize voxel-based analysis of FA or MRE to compare between an individual patient and a group of healthy controls.^34^

There are several limitations of this feasibility study. Along with a small sample size, there was additional selection bias within this sample, as participants with the most severe TBI participating in acute rehabilitation were sometimes not sufficiently stable to undergo MRI (e.g., post-traumatic agitation, acute medical complications). Although all individuals had a moderate-severe TBI requiring acute inpatient rehabilitation, those that consented for participation in the study had relatively milder injuries as measured by duration of PTA (mean 7.9 days), and all recovered well based on the GOSE, FIM, and DRS outcomes at 1 year. Additionally, a conscious decision was made to exclude individuals who had undergone prior intracranial procedures from MRE in this preliminary investigation. Although this exclusion was prudent for the initial scope of our feasibility study, further inquiry is necessary to understand the safety and applicability of MRE in that group of individuals. This limitation indeed makes the interpretation of our results challenging in representing those with more severe TBI. These factors limited our ability to detect statistical significance within MRE data, and also hindered our ability to explore and develop a more comprehensive model that would encompass conventional MRI, DTI, and MRE. In this regard, our interpretations should be approached with caution. Despite these constraints, a significant correlation between hemorrhage scores and PTA duration was identified, along with promising trends linking the lesion-to-normal stiffness ratio with PTA durations. These findings suggest the potential utility of these imaging-based biomarkers for predicting outcomes in a broader clinical cohort.

## Conclusion

This feasibility study is the first to report MRE in relation to clinical indicators of injury severity, incorporating both conventional MRI and DTI, as well as MRE. Future larger studies with a wider range of injury severity and including individuals requiring intracranial procedures could lead to the development of prediction models integrating anatomical (MRI), structural (DTI), and mechanical (MRE) changes caused by moderate-severe TBI. Such a prediction model would use imaging and clinical data available in the acute phase after injury to inform prognosis and care planning.

## Supplementary Material

Supplemental data

## Abbreviations Used

CT: computed tomography
DTI: diffusion tensor imaging
FA: fractional anisotropy
FLAIR: fluid attenuated inversion recovery
MRE: magnetic resonance elastography
MRI: magnetic resonance imaging
NDB: national data base
PTA: post-traumatic amnesia
TBI: traumatic brain injury
TFC: time to follow commands
TBIMS: traumatic brain injury model system center (TBIMS)
WM: white matter
