# Supplementary material for: Association Between Anatomic and Clinical Indicators of Injury Severity After Moderate-Severe Traumatic Brain Injury: A Pilot Study Using Multiparametric Magnetic Resonance Imaging
Source: Neurotrauma Rep. 2024 Mar 13;5(1):232–42. doi: 10.1089/neur.2023.0122 (PMC10960168; doi:10.1089/neur.2023.0122)
Supplement: Supplementary file 1 [file Suppl_Data.odt]

Supplementary Tables

Table 1: Non-hemorrhagic contusion score characterization (lesion volume was characterized by visual inspection).

| FLAIR Number of lesions |  |  | Patient #1 | Patient #2 | Patient #3 | Patient #4 | Patient #5 | Patient #6 | Patient #7 | Patient #8 | Patient #9 |
| --- | --- | --- | --- | --- | --- | --- | --- | --- | --- | --- | --- |
| Pt score |  |  | 3 | 2 | 2 | 2 | 1 | 3 | 1 | 2 | 2 |
| Hemispheres | Frontoinsular | Right | 3 | 1 | 1 |  |  | 5 |  | 1 | 1 |
| Left |  |  |  |  |  | 3 |  | 2 | 3 |
| Temporal | Right | 2 |  |  |  |  | 1 |  |  |  |
| Left |  |  |  |  |  |  |  |  |  |
| Parietooccipital | Right | 3 |  | 1 |  |  |  |  |  |  |
| Left |  |  |  | 1 |  |  |  |  |  |
| Deep brain structures | Lentiform nucleus | Right |  |  |  |  |  |  |  |  |  |
| Left |  |  |  |  |  |  |  |  | 1 |
| Caudate nucleus | Right |  |  |  |  |  |  |  |  | 1 |
| Left |  |  |  |  |  |  |  |  |  |
| Thalamus | Right |  |  |  |  |  |  |  |  |  |
| Left |  |  |  |  |  |  |  |  |  |
| Internal capsule | Right |  |  |  |  |  |  |  |  |  |
| Left |  |  |  |  |  |  |  |  |  |
| Corpus callosum | Genu |  |  |  |  |  |  |  |  | 1 |
| Body |  |  |  |  |  |  |  |  |  |
| Splenium |  | 1 |  |  |  |  |  |  |  |
| Brainstem and cerebellum | Cerebral peduncles and midbrain | Right |  |  |  |  |  |  |  |  |  |
| Left |  |  |  |  |  |  |  |  |  |
| Pons and medulla | Right |  |  |  |  |  |  |  |  |  |
| Left |  |  |  |  |  |  |  |  |  |
| Cerebellum | Right |  |  |  |  |  |  |  |  |  |
| Left |  |  | 2 (1 is vermis) |  |  |  |  |  |  |

Table 2: Hemorrhage score characterization.

| SWAN Number of microhemorrhages |  |  | Patient #1 | Patient #2 | Patient #3 | Patient #4 | Patient #5 | Patient #6 | Patient #7 | Patient #8 | Patient #9 |
| --- | --- | --- | --- | --- | --- | --- | --- | --- | --- | --- | --- |
| Pt score |  |  | 4 | 3 | 3 | 1 | 2 | 4 | 2 | 4 | 4 |
| Hemispheres | Frontoinsular | Right | 15 | 2 |  |  |  | 43 |  | 27 | 6 |
| Left | 4 | 3 |  |  | 1 | 6 |  | 19 | 8 |
| Temporal | Right | 23 |  | 3 |  | 2 | 33 |  | 8 | 4 |
| Left | 5 | 1 |  |  |  | 10 |  |  |  |
| Parietooccipital | Right | 11 |  | L-1 |  |  | 7 |  |  |  |
| Left | 5 |  |  |  |  | 3 |  | 2 |  |
| Deep brain structures | Lentiform nucleus | Right |  |  | 3 |  |  |  |  |  |  |
| Left |  |  | 1 |  |  | 1 |  |  |  |
| Caudate nucleus | Right |  |  |  |  |  |  |  | 3 |  |
| Left |  |  |  |  |  |  |  |  |  |
| Thalamus | Right |  |  |  |  |  | 3 |  |  |  |
| Left |  |  |  |  |  |  |  |  |  |
| Internal capsule | Right |  |  |  |  |  |  |  | 1 |  |
| Left |  |  |  |  |  | 1 |  |  |  |
| Corpus callosum | Genu |  |  | 1 |  |  | 2 |  | 5 |  |
| Body |  |  |  |  |  | 3 |  | 1 |  |
| Splenium |  |  | 1 |  |  |  |  |  |  |
| Brainstem and cerebellum | Cerebral peduncles and midbrain | Right |  |  | 1 |  |  | 4 |  |  |  |
| Left |  | 1 | 3 |  |  | 1 |  |  |  |
| Pons and medulla | Right |  |  |  |  |  |  |  |  |  |
| Left |  |  |  |  |  | 2 |  |  |  |
| Cerebellum | Right |  |  |  |  |  |  |  | 3 |  |
| Left |  | 1 |  |  |  |  | 1 | 5 |  |

Supplemental data – description regarding imaging characteristics and MRI/MRE analyses

Imaging characteristics included MRI-assessed non-hemorrhage contusion score, MRI-assessed hemorrhage score, DTI measured White Matter (WM) fractional anisotropy (FA), and MRE measured lesion stiffness. 

The MRI included the following: 1) a 3D T1-weighted (T1W) image acquired using a magnetization-prepared rapid gradient echo (MP-RAGE) pulse sequence with: repetition time (TR)/echo time (TE)  = 7.4/3.0 ms, flip angle = 8°, inversion time (TI) = 900 ms, field of view (FOV) = 26 cm; acquisition matrix = 256 x 256, and 166 slices with a spacing of 1.2 mm; (2) an axial T2-weighted (T2W) image acquired with fast spin echo (FSE) pulse sequence with: TR/TE = 6285/103 ms, echo train length = 12, flip angle = 111°, FOV = 22 cm, 46 axial slices, slice thickness = 3 mm; (3) an axial T2 FLAIR with: TR/TE = 11000/150 ms, TI = 2250 ms, flip angle = 125°, FOV = 22 cm, 46 axial slices, slice thickness = 3 mm; (4) an axial susceptibility-weighted angiography (SWAN) image acquired with: TR/TE = 37.4/23.6 ms; flip angle = 15°; FOV = 22 cm, slice thickness = 2 mm; and (5) a diffusion tensor imaging (DTI) scan acquired using single-shot spin-echo echoplanar imaging (SE-EPI) with: 48 diffusion directions with b = 800 s/mm2, 6 images with b = 0 s/mm2, TR/TE = 4600/63.1 ms, FOV = 24 cm, matrix = 128 × 128, slice thickness = 3 mm, 48 axial slices.

MRE scans involved the application of 60Hz micrometer-level vibrations, generated by a commercially available pneumatic active driver (Resoundant Inc., Minnesota, USA). These vibrations were transmitted through a custom-made, soft, pillow-like head passive driver positioned beneath each participant's head to induce motion within the intracranial brain tissue (Figure 1). The resulting brain motions were captured using a single shot, spin-echo (SE), echo-planar imaging (EPI) based dual-sensitivity motion encoding MRE pulse sequence1 with the following parameters: TR/TE = 4000/58.7 ms, FOV = 240 x 240 mm, matrix size = 80×80, 48 contiguous 3-mm-thick axial slices, 2xASSET acceleration; 3 phase offsets sampled over one period of excitation. The full 3D displacement field was recorded by employing the motion encoding gradients (MEG) along the ±x- (frequency encoding, left-to-right), ±y- (phase encoding, anterior-to-posterior), and ±z- (slice selection, superior-to-inferior) directions.

MRI MRE quantitative analysis

The preprocessing of the diffusion data for DTI scan involved employing a previous-developed pipeline,2 encompassing procedures such as skull stripping, denoising, correction for head motion, eddy current distortions, Gibbs ringing, and Rician noise bias. Subsequently, the FA map was derived from the diffusion tensors by applying a nonlinear least square fitting algorithm.3 A standard TBSS pipeline implemented in the FMRIB software library (FSL v6.0, https://fsl.fmrib.ox.ac.uk/fsl/) was used for the mean FA calculation. The FA maps in native space were nonlinearly registered to the standard space (FMRIB58_FA template), and then the mean FA image of all subjects was generated and thinned to create a mean FA skeleton. A threshold of 0.2 was used for the mean FA skeleton image. The global mean FA of the white matter skeleton was finally reported for each subject. Considering the lack of a control dataset, control of aging-related effects was achieved by referencing published outcomes of a linear regression analysis.4

The maps of brain stiffness were computed using a previously published neural network inversion algorithm.5 The neural network is trained using simulated inhomogeneous wave patches generated through a finite difference model. The properties of the simulated material are randomly selected, with stiffness values ranging from 1 to 10 kPa and damping ratios ranging from 0 to 0.5 in order to match the in-vivo data. Each simulated wave patch is augmented with a randomly selected in-vivo brain mask patch and complex-valued zero-mean Gaussian noise. An Inception-like neural network architecture is trained using the TensorFlow 2.0 platform, employing a mean squared error loss function. To ensure the accuracy and reliability of MRE measurements, the mean stiffness values were reported based on the region of interest that corresponds to non-hemorrhagic contusion lesions larger than 100 pixels. The FLAIR images were used to generate two regions of interest: lesion and contralateral normal brain. The FLAIR images along with the lesion/control masks were registered and resliced to the MRE space. Trilinear interpolation was used for resampling the anatomical images, while the lesion/control mask was resampled with nearest-neighbor interpolation. To account for any mechanical behavior not specifically due to brain trauma, we calculated the ratio of lesion stiffness to the contralateral normal brain tissue stiffness. For participants with multiple lesions, the largest ratio of lesion/normal stiffness among all the lesions was reported. If no lesions were identified from the FLAIR images, the ratio of lesion/normal stiffness was set as 1, assuming that the material properties were the same on both sides.

1.	Yin Z, Sui Y, Trzasko JD, et al. In vivo characterization of 3D skull and brain motion during dynamic head vibration using magnetic resonance elastography. Magn Reson Med 2018;80(6):2573-2585. (In eng). DOI: 10.1002/mrm.27347.

2.	Raghavan S, Przybelski SA, Reid RI, et al. Reduced fractional anisotropy of the genu of the corpus callosum as a cerebrovascular disease marker and predictor of longitudinal cognition in MCI. Neurobiology of Aging 2020;96:176-183. DOI: [https://doi.org/10.1016/j.neurobiolaging.2020.09.005](https://doi.org/10.1016/j.neurobiolaging.2020.09.005).

3.	Garyfallidis E, Brett M, Amirbekian B, et al. Dipy, a library for the analysis of diffusion MRI data. Frontiers in Neuroinformatics 2014;8 (Methods) (In English). DOI: 10.3389/fninf.2014.00008.

4.	Inano S, Takao H, Hayashi N, Abe O, Ohtomo K. Effects of age and gender on white matter integrity. AJNR Am J Neuroradiol 2011;32(11):2103-9. (In eng). DOI: 10.3174/ajnr.A2785.

5.	Murphy MC, Cogswell PM, Trzasko JD, et al. Identification of Normal Pressure Hydrocephalus by Disease-Specific Patterns of Brain Stiffness and Damping Ratio. Invest Radiol 2020;55(4):200-208. (In eng). DOI: 10.1097/rli.0000000000000630.
